# Electrophysiological frequency domain analysis of driver passive fatigue under automated driving conditions

**DOI:** 10.1038/s41598-021-99680-4

**Published:** 2021-10-13

**Authors:** Yijing Zhang, Jinfei Ma, Chi Zhang, Ruosong Chang

**Affiliations:** 1 School of Psychology, Liaoning Normal University, Dalian, 116029 China; 2 School of Biomedical Engineering, Faculty of Electronic Information and Electrical Engineering, Dalian University of Technology, Dalian, 116024 China

**Keywords:** Psychology, Human behaviour

## Abstract

With the continuous improvement of automated vehicles, researchers have found that automated driving is more likely to cause passive fatigue. To explore the impact of automation and scenario complexity on the passive fatigue of a driver, we collected electroencephalography (EEG), detection-response task (DRT) performance, and the subjective report scores of 48 drivers. We found that in automated driving under monotonic conditions, after 40 min, the alpha power of the driver’s EEG indicators increased significantly, the accuracy of the detection reaction task decreased, and the reaction time became slower. The receiver characteristic curve was used to calculate the critical threshold of the alpha power during passive fatigue. The determination of the threshold further clarifies the occurrence time and physiological characteristics of passive fatigue and improves the passive fatigue theory.

## Introduction

Mental fatigue is one of the important risk factors of traffic accidents worldwide^1–3^, accounting for about 20% of traffic accidents^4–6^. Mental fatigue can be divided into active fatigue and passive fatigue^7–9^. Active fatigue is caused by tasks that require continuous coordination of perceptual activity. Passive fatigue is caused by tasks requiring few perceptual activities and long-term monotonous reactions. Some researchers proposed that the key to dividing both fatigues depends on mental workload^7^. The fatigue induced by low mental loads is passive, and the fatigue caused by high mental loads is active. Studies generally classify these by creating driving scenarios with different complexity levels^8–12^. Complex driving scenarios have a cumulative effect on mental workload^13–16^.

With the gradual improvement of vehicle automation, drivers free from driving tasks are more prone to lower mental workload conditions^17^. De Winter et al.^18^ summarised 32 empirical studies on drivers’ mental workload. They established that the mental workload of drivers under automated driving was reduced by 20.8% compared to manual driving. Therefore, this study quantifies the co-variation relationship between mental workload and passive fatigue by creating traffic scenarios with different complexity levels and driving models.

A driver’s mental workload underload may induce passive fatigue states^14,16^. Based on the eye movement index, Körber et al.^8^ found that drivers experienced fatigue in about 42 min, indicating that it took a specific time for drivers to develop a low mental workload from fatigue. Vogelpohl et al.^19^ proved this by judging the time to the development of fatigue under automatic driving conditions to be 15–35 min based on facial expressions.

There are many ways to measure driving fatigue. When driving manually, researchers often use methods such as subjective reports and vehicle speed variability^2^. During automation, the speed of drivers with passive fatigue became slower with regard to responding to takeover requests^20–22^; in addition, more frequent involvement of non-driving-related tasks occurred^23^. These indicators can sensitively reflect fatigue in practice; however, the driver’s state is not sufficient to measure fatigue even if monitored accurately and in real time. Researchers favour EEG indicators due to their accuracy and real-time performance^24^. Lal et al.^2^ used the average EEG activity of waking state participants as a fatigue benchmark. They analysed the characteristics of the changes in the participants’ EEG results while in different fatigue stages and concluded that when the driver was fatigued, their delta and theta activity increased. In a simulated driving scenario, Jagannath and Balasubramanian^11^ found that as the test participants’ fatigue increased, the alpha power increased significantly and theta power decreased significantly; however, to accurately define a driver’s passive fatigue state, a clearer definition standard is required. Thus, the primary goal of this research was to analyse the time-domain characteristics of EEG signals in different states and select the alpha power as a passive indicator. Further, we used the receiver operating characteristic curve (ROC) analysis method to determine the driver’s passive fatigue discrimination threshold. Therefore**,** we proposed the following hypotheses.

### **Hypothesis 1**

A driver’s mental workload in automated driving under monotonic conditions is small.

### **Hypothesis 2**

A low mental workload when driving will take approximately 40 min to induce the driver to experience passive fatigue.

### **Hypothesis 3**

Based on the ROC curve method, the alpha power critical threshold of the driver's passive fatigue and waking state can be calculated.

## Methods

### Experimental design

A three-factor, 2 (driving mode: autopilot and manual driving) × 2 (driving condition: monotonous and engaging conditions) × 6 (measurement stage: 1–6) experiment was designed for this study. The driving mode and driving conditions were the between-subject variables. Taking into account the stage and accumulation of fatigue, EEG and detection response tasks were divided into six stages^25^: Stages 1–6 correspond to data acquired from the first 0–10 min, 10–20 min, 20–30 min, 30–40 min, 40–50 min, and 50–60 min, respectively. The six stages of driving were the within-subject variables.

### Participants

From August to October 2020, we recruited 48 participants aged 20–35 years (*M* = 24.83, *SD* = 2.81) with no experience using automated vehicles and a driving history of 1–10 years (*M* = 2.94, *SD* = 2.06). Among them, there were 24 men and 24 women. All participants were randomly divided into 4 groups (12 people per group). All participants were in good health with normal hearing and eyesight. To avoid the influence of physical fatigue, the experiment time was set from 9:00 to 11:00 every morning^26^; the participants were required to get enough sleep the night before, and they were not allowed to drink coffee, alcohol, or tea 24 h before the experiment. After the experiment, a test fee of RMB 100 was given. This study was approved by the Ethics Committee of Liaoning Normal University and was performed in accordance with the approved guidelines and the Declaration of Helsinki. All the participants provided written informed consent before participating and knew that their identifying images will published in an online open-access journal.

### Equipment

#### Driving simulator

**Figure 1:**
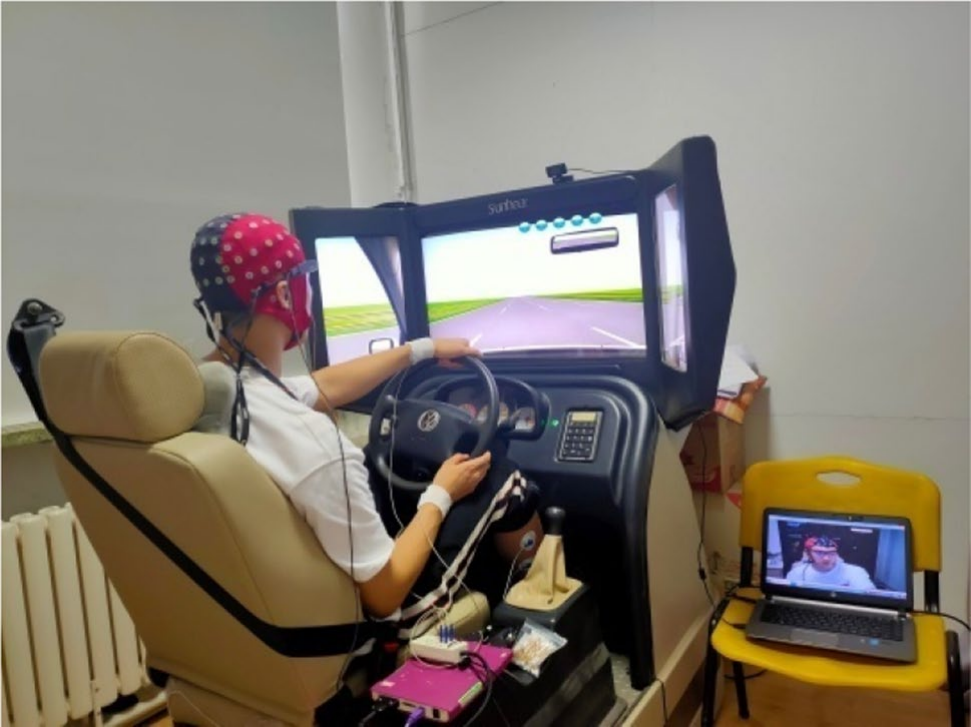
Automated driving scenario in the Sunheart QJ-3A1 Driving Simulator (small).

The detection-response task (DRT) was performed using a Xuan’ai QJ-3A1 (small) driving simulator with a viewing angle of 120°. The simulator consists of an interactive visual system, a simulated cockpit, an electronic control system, customised software, auxiliary equipment, and exterior parts. It has functions such as video teaching, guided driving simulation exercises, interactive scene experience driving, and accident tendency assessments, as shown in Fig. 1.

The simulator can design the randomly appearing picture stimuli on the left and right sides as a detection-response task, with a time interval of 60 ± 40 s. The reaction time from the appearance of the picture stimulus to the driver’s braking was automatically recorded. After the experiment was completed, the number of trials that exceeded 2 s during the reaction were recorded as errors for which to evaluate the driver’s correct rate.

The driving modes and scenarios were generated using a driving simulator. In the automated driving mode, the driver does not need to operate the steering wheel and accelerator pedal; they only need to press the brake in response to the DRT. In the manual driving mode and the brake response to the DRT condition, the driver also needs to control the vehicle to drive along the established route. The engaging driving conditions, which the driver used for 1 h, included cities, towns, tunnels, high-speed complex roads, and multi-curved roads. The monotonous driving scene was a straight, monotonous highway with no buildings on either side.

#### EEG

**Figure 2:**
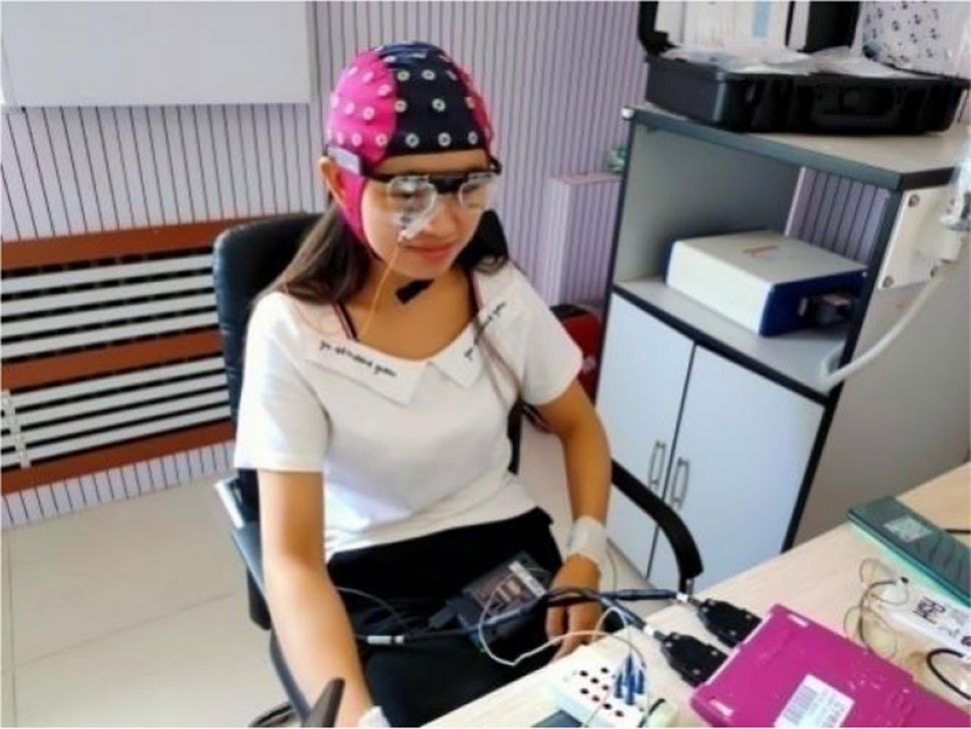
EEG data collection scene.

EEG data were recorded using 64 Ag–AgCl scalp electrodes placed according to the International 10–20 system (ANT Neuro; pass band: 0.01–100 Hz; sampling rate: 1000 Hz). The Cpz was used as the reference, and electrode impedances were kept lower than 10 kΩ, as shown in Fig. 2.

#### NASA-Task load index

The NASA-Task Load Index (NASA-TLX), developed by the National Aeronautics and Space Administration^27^, is a scale that incorporates mental demand, physical demand, effort, temporal demand, performance, and frustration in six dimensions. Each dimension is divided into 10 levels from low to high, where “1” means that the status is extremely low and “10” means that the status is extremely high. The NASA-TLX is an effective tool for measuring mental workload and has been applied in several studies^28^. In this study, we use it to measure the driver’s mental workload.

#### SOFI

The Swedish Occupational Fatigue Inventory-25 (SOFI), compiled by Ahsber et al.^29^, consists of 25 questions divided into five dimensions: lack of energy, physical exertion, physical discomfort, lack of motivation, and drowsiness. There are five questions in each dimension, and each topic from 0 to 10 is divided into 11 levels: 0 represents a very small fatigue state level, and 10 represents an extremely large fatigue state level. In this study, we use it to measure the driver’s fatigue.

### Procedure

The experiment lasted for 1 h. After participants arrived in the laboratory, they were asked to wash their hair and to wear an EEG cap and eye tracker. Before starting the experiment, the participants were required to familiarise themselves with the driving simulator and practice three random DRT (approximately 5 min) tasks. Further, they were required to fill in the NASA-TLX and SOFI scales as pre-tests. After the experiment was officially started, the participants were asked to fill in the NASA-TLX every 10 min. To fill in the forms, the driver pulled over and we stopped recording the EEG and DRT data. After the experiment, the participants were required to fill in the NASA-TLX and SOFI post-tests.

### EEG data preprocessing

According to the principle of resting EEG data preprocessing^30^, after filtering the continuous EEG data between 0.5 and 30 Hz, the EEG data of each participant during the simulated driving process were divided into six parts, corresponding to stage 1–6, respectively. Each phase lasted about 10 min, and the sampling rate was reduced to 250 Hz. EEG data were referenced to the average of both mastoids (M1, M2). The Independent Component Analysis (ICA) algorithm was used to correct the part of the data contaminated by eye movement or electromyography (EMG) data or by any other non-physiological diseases.

### Power computation

For each participant, the pre-processed continuous EEG data were segmented into dozens of epochs, with epoch length of 2000 ms. Then the 61-channel segmented epochs were transformed to the frequency domain based on Fast Fourier transforms (FFTs) using a Hamming window with a 50% overlap, yielding FFTs ranging from 0.5 to 30 Hz with a frequency resolution of 0.5 Hz^31^. The power spectrum of each frequency point was averaged over the epochs. Previous studies indicated that EEG algorithm alphas showed larger increases as fatigue increased^10^. The power of alpha bands were the largest in parietal lobe and the power of each band was distributed symmetrically between the left hemisphere and the right hemisphere^25^. Therefore, this study selected the P3, Pz, P4 electrode data to implement the difference tests. Single-subject EEG spectra were averaged across subjects in each group in order to obtain group-level EEG spectra.

### Data analysis

According to previous studies^25^, the present data (subjective reports, DRT performance, and alpha power) were subjected to 2 (driving mode: autopilot and manual driving) × 2 (driving condition: monotonous and engaging conditions) × 6 (stage 1–6) three-factor repeated measurement analysis.

## Results

### Subjective report

#### NASA-TLX

With the driving mode and scenario complexity as the between-subject variables, the measurement stage as the within-subject variable, and the cognitive load dimension score as the dependent variable, a repeated measures ANOVA was performed. The main effect of scenario complexity was significant [*F*(1, 44) = 4.890, *p* = 0.046, *η*_*p*_^2^ = 0.088]. Drivers in the engaging condition (4.96 ± 2.116) felt a higher psychological load than the simple scenario group (2.04 ± 2.000).

With the driving mode and scenario complexity as the between-subject variables, the measurement stage as the within-subject variable, and the frustration dimension score as the dependent variable, a repeated measures ANOVA was performed. The main effect of the measurement stage was significant [*F*(1, 44) = 7.537, *p* = 0.009, *η*_*p*_^2^ = 0.146]. As the measurement stage increased, the driver’s degree of frustration also increased. The main effects of the driving mode and scenario complexity were insignificant, and the interaction effects were also insignificant. No other main effects or interaction effects were significant in other dimensions.

#### SOFI

With the driving mode and scenario complexity as the between-subject variables, the measurement stage as the within-subject variable, and the lack of energy physical exertion, physical discomfort, lack of motivation, and drowsiness dimensions as the dependent variables respectively, a repeated-measures ANOVA was performed. The main effect of the measurement stage in five dimensions was significant (Table 1). As the measurement stage increased, the driver’s degree of lack of energy also increased. The main effects of the driving mode and scenario complexity were insignificant, and the interaction effects were also insignificant in all dimensions.

**Table 1:** Analysis of the difference between the five dimensions of the SOFI before and after the test.

Dimension	Before	After	*F*	*p*
Lack of energy	1. 386 ± 1.887	3.390 ± 2.486	21.909	0.000
Physical exertion	1.251 ± 1.401	1.907 ± 1.532	6.034	0.019
Physical discomfort	1.209 ± 1.359	1.879 ± 1.461	7.969	0.008
Lack of motivation	1.688 ± 1.800	3.237 ± 2.368	39.000	0.000
Sleepiness	1.688 ± 2.000	3.967 ± 2.789	27.586	0.000

### DRT performance

#### Reaction time of the DRT

With the driving mode and scenario complexity as the between-subject variables, the measurement stage as the within-subject variable, and the reaction time of the DRT as the dependent variable, a repeated measures ANOVA was performed. The results indicated an insignificant main effect of scenario complexity [*F*(1, 44) = 0.185, *p* = 0.996, *η*_*p*_^2^ = 0.004] and driving mode [*F*(1, 44) = 2.303, *p* = 0.136, *η*_*p*_^2^ = 0.336]. There was a significant interaction between driving mode and measurement stage [*F*(2, 88) = 3.851, *p* = 0.025, *η*_*p*_^2^ = 0.080].

**Figure 3:**
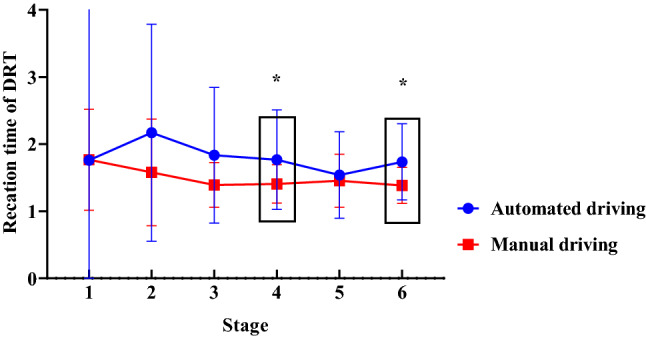
Influence of driving mode on the reaction time of the detection reaction task in the six stages (Note: *p < 0.05).

The simple effect test showed that the difference in the DRT reaction time caused by the driving mode was insignificant in stage 1 [*F*(1, 44) = 0.000, *p* = 0.989, *η*_*p*_^2^ = 0.000], stage 2 [*F*(1, 44) = 2.631, *p* = 0.112, *η*_*p*_^2^ = 0.056], stage 3 [*F*(1, 44) = 4.003, *p* = 0.052, *η*_*p*_^2^ = 0.083], and stage 5 [*F*(1, 44) = 0.297, *p* = 0.589, *η*_*p*_^2^ = 0.007]; in stages 4 and 6, the difference in the DRT reaction time as a result of the driving mode was significant [*F*(1, 44) = 4. 856, *p* = 0.033, *η*_*p*_^2^ = 0.099; *F*(1, 44) = 7.456, *p* = 0.009, *η*_*p*_^2^ = 0.145]. In addition, the reaction time of the driver during automated driving (1.768 ± 0.740; 1.735 ± 0.567) was higher than that of manual driving (1.406 ± 0.285; 1.383 ± 0.267), as shown in Fig. 3.

#### DRT accuracy

With the driving mode and scenario complexity as the between-subject variables, the measurement stage as the within-subject variable, and the DRT accuracy as the dependent variable, a repeated measures ANOVA was performed. The results indicated an insignificant main effect of scenario complexity [*F*(1, 44) = 0.185, *p* = 0.996, *η*_*p*_^2^ = 0.004] and driving mode [*F*(1, 44) = 2.303, *p* = 0.136, *η*_*p*_^2^ = 0.336]. There was a significant interaction between the driving mode and measurement stage [*F*(2, 88) = 3.851, *p* = 0.025, *η*_*p*_^2^ = 0.080].

**Figure 4:**
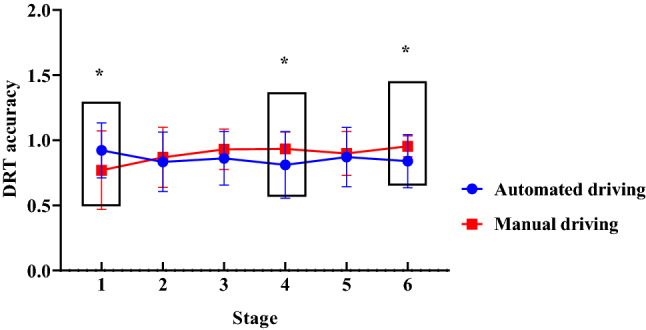
The influence of driving mode on the DRT accuracy in the six stages (Note: *p < 0.05).

The simple effect test showed that the difference in DRT accuracy caused by the driving mode was insignificant in stage 2 [*F*(1, 44) = 0.281, *p* = 0.599, *η*_*p*_^2^ = 0.006], stage 3[*F*(1, 44) = 1.703, *p* = 0.198, *η*_*p*_^2^ = 0.036], and stage 5 [*F*(1, 44) = 0.243, *p* = 0.624, *η*_*p*_^2^ = 0.005]; in stages 1, 4, and 6, the difference in DRT accuracy as a result of the driving mode was significant [*F*(1, 44) = 4.805, *p* = 0.049, *η*_*p*_^2^ = 0.082]; *F*(1, 44) = 4. 468, *p* = 0.040, *η*_*p*_^2^ = 0.089; *F*(1, 44) = 6.491, *p* = 0.014, *η*_*p*_^2^ = 0.124]. For stages 4 and 6, the DRT accuracy of the driver in the automated driving condition (0.811 ± 0.256; 0.840 ± 0.204) was lower than that of the manual driving condition (0.934 ± 0.129; 0.954 ± 0.080), as shown in Fig. 4.

### EEG

#### Brain topography

**Figure 5:**
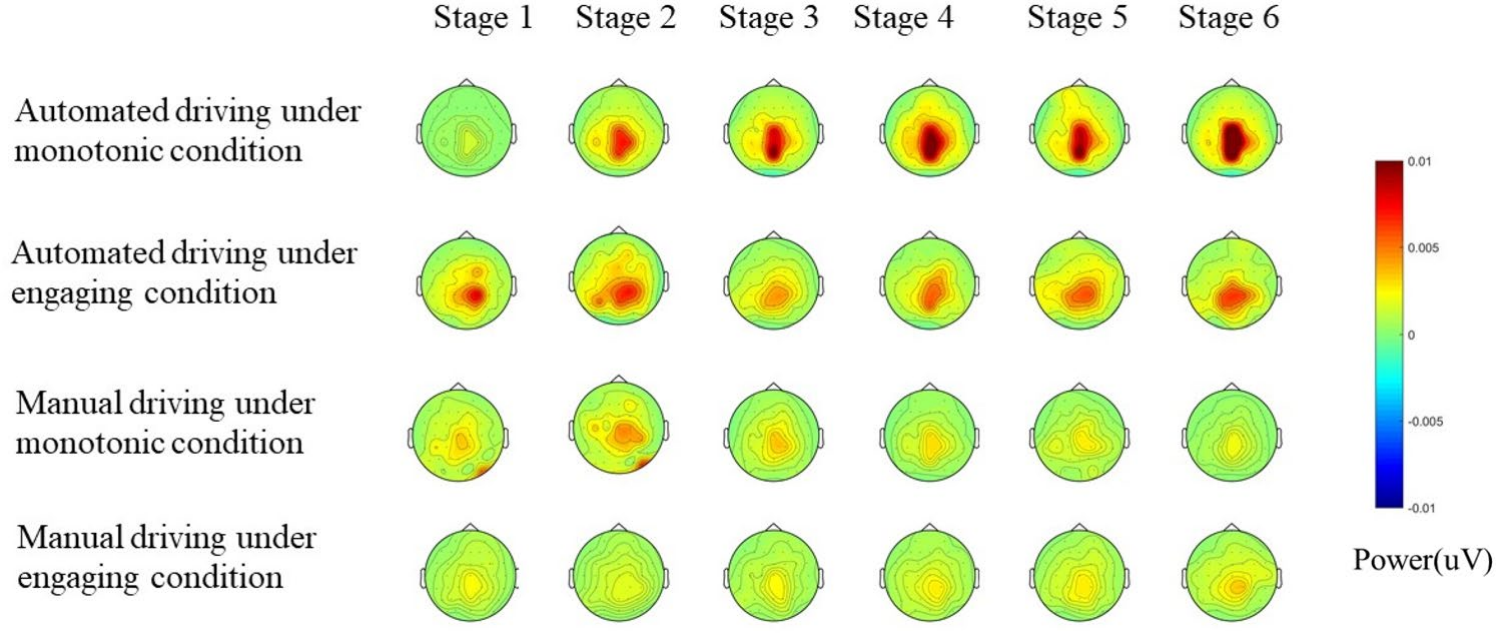
Map of the alpha brainwaves of the average of all drivers.

The single-subject EEG spectra were averaged across subjects in each group in order to obtain the group-level EEG spectra. The alpha (8–13 Hz) power topographies were displayed as four groups and six stages (Fig. 5).

#### Alpha power

With the driving mode and scenario complexity as the between-subject variables, the measurement stage as the within-subject variable, and the α power value as the dependent variable, a repeated measures ANOVA was performed. The results indicated an insignificant main effect of scenario complexity [*F*(1, 37) = 0.223, *p* = 0.640, *η*_*p*_^2^ = 0.006] and driving mode [*F*(1, 37) = 3.192, *p* = 0.082, *η*_*p*_^2^ = 0.079]. There was a significant interaction between the driving mode, scenario complexity, and measurement stage [*F*(1, 37) = 4.651, *p* = 0.038, *η*_*p*_^2^ = 0.112].

**Figure 6:**
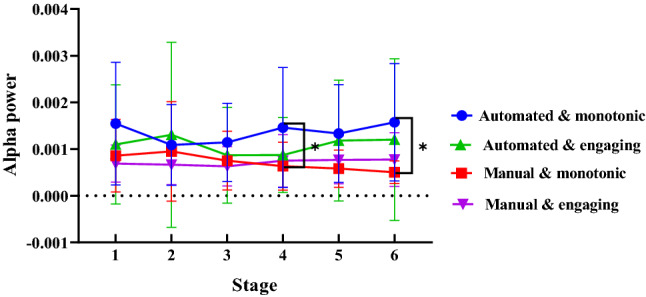
Interaction of driving modes, driving scenarios, and driving stages on α power (Note: *p < 0.05).

The simple effect test shows that under complex scenarios, the differences in the driving modes in the six stages were insignificant. In the simple scenario, there was no significant difference in the power of the alpha power in stage 1 [*F*(1, 37) = 4.651, *p* = 0.038, *η*_*p*_^2^ = 0.026], stage 2 [*F*(1, 37) = 4.651, *p* = 0.038, *η*_*p*_^2^ = 0.005], stage 3 [*F*(1, 37) = 4.651, *p* = 0.038, *η*_*p*_^2^ = 0.018], and stage 5 [*F*(1, 37) = 4.651, *p* = 0.038, *η*_*p*_^2^ = 0.04]. In stages 4 and 6, the driving mode has a significant effect on the power of the alpha value [*F*(1, 37) = 4.651, *p* = 0.038, *η*_*p*_^2^ = 0.062; *F*(1, 37) = 4.651, *p* = 0.038, *η*_*p*_^2^ = 0.015]. As shown in Fig. 6, in stages 4 and 6 of the simple scenario, the alpha power of the automatic driving group was significantly higher than that of the manual driving group.

#### Judging the critical threshold based on the ROC curve

**Figure 7:**
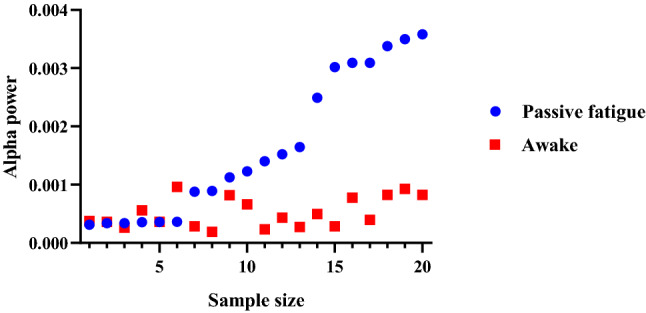
Scatter plot before and after passive fatigue.

The data of stages 4 and 6 of manual driving under monotonic conditions were regarded as the awake group, and the data of stages 4 and 6 of automated driving under monotonic conditions driving were regarded as the passive fatigue group. A scatter diagram of the alpha power of the EEG signals in the awake and passive fatigue states is shown in Fig. 7.

Using GraphPad Prism 8 software to perform ROC calculations on two sets of data, the results showed a significant difference (*p* = 0.021) between the alpha power of the EEG signals of the awake and passive fatigue states, indicating that the alpha power reflects the fatigue state of drivers and can be used as a discrimination index of passive fatigue.

**Figure 8:**
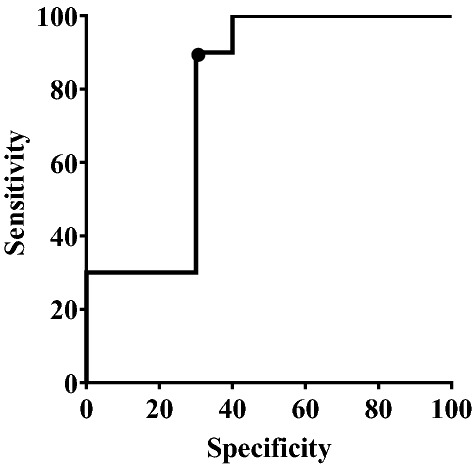
ROC curve of EEG alpha power.

To determine the judgement threshold of passive fatigue, the ROC curve based on the alpha power was drawn according to the ROC curve analysis method, as shown in Fig. 8. According to the principle of threshold selection, the feature point with the largest index in the upper left of y was selected as the discrimination threshold. The y index corresponding to each discrimination threshold was calculated, and the feature point with the largest index (the point in Fig. 8) was selected as the best critical point, corresponding to the EEG signal; the alpha power was 0.000852, sensitivity was 90%, and specificity was 70%. The area under the ROC curve was 0.78, indicating that the method was highly accurate.

## Discussion

This study aimed to examine the passive fatigue of driver under automation. Our behavioural results confirmed that driver DRT performance begins to decline at 40 min. This result fits well with the EEG data. The power value of the alpha was also significant in the other groups after 30–40 min and 50–60 min. We believe this is indicative of passive fatigue occurring in the driver.

It should be noted that the EEG power in this study is low. It may be caused by the calculation method of power. In the research using the same power calculation method, the beta frequency band varies in the range of 0.0005–0.007, which is not high either^31,32^. Therefore, even if the value of Alpha power is low (basically between 0.0013 and 0.0025), we still report them objectively.

Most current studies involving passive fatigue rely only on the one-way dimension of driving situational needs to define low-load conditions^33–35^. Our study confirmed the impact of driving scenarios on the driver’s mental workload under autonomous driving conditions; in the automatic driving mode, monotonous scenarios would lower the mental workload. Therefore, the induction of passive fatigue in the future should be multi-dimensional, making it possible to guarantee experimental validity induced by passive fatigue. According to the definition of passive fatigue^7^, the key to distinguishing between active and passive fatigue is mental workload. We found that passive fatigue induction was successful only when the experimental participants had a lower mental workload with accumulated fatigue.

In this study’s subjective report, the driver’s mental workload in the monotonic state was lower than that in the engaging condition. The drivers in the simple scenarios experienced more fatigue. This shows that the drivers’ fatigue in this study belonged to a passive fatigue state. The subjective report further found that the driver was physically exerted at this time, mainly experiencing fatigue symptoms such as breathing heavily, feeling out of breath, tasting blood, sweating, and experiencing heart palpitations. This result reminds us that the user experience of autonomous driving functions needs to be urgently improved in simple situations. In the future, researchers should continue to explore the regulation and improvement of drivers’ passive fatigue, develop multiple forms of vigilance maintenance tasks, and maintain the driver’s mental load level in the optimal load zone to avoid the generation of passive fatigue symptoms.

As the subjective report is only filled out before and after the experiment, the precise time of passive fatigue cannot be determined. Through further analysis of the data, it was found that in automated driving under monotonic conditions, the EEG indicators and the performance of the detection response task were significantly different after around 40 min of manual driving under monotonic conditions. Therefore, we believe that drivers will experience passive fatigue in the simple scenario of automatic driving after around 40 min. However, the time required to induce passive fatigue differs under various scenarios. Thus, time cannot be used as the gold standard for evaluating passive fatigue.

It is necessary to quantify the physiological characteristics of a driver’s passive fatigue. After clarifying the occurrence time of passive fatigue, we analysed the alpha power of the drivers during automated driving under monotonic conditions and during manual driving under monotonic conditions. Using the ROC curve method, the threshold of passive fatigue discrimination based on the alpha power was 0.000852. The determination of the threshold further clarifies the occurrence time and physiological characteristics of passive fatigue and improves the passive fatigue theory.

Finally, whether it is the performance of DRT or the driver’s EEG indicators, at a stage after the emergence of passive fatigue, the differences caused by passive fatigue disappeared; this seems to be a type of regression. We believe that this regression occurs because the driver adapts to the fatigue state through subjective regulation. The driver’s regulation can be divided into two types: self-regulation and external regulation. In self-regulation, the driver adjusts their cognitive strategy, optimises their processing mode, makes psychological efforts, actively adjusts their mental workload, and improves their passive fatigue state. In external regulation, drivers participate in external activities more frequently, seeking an increase in task demands. At present, many studies have proved the existence of external regulation. Naujoks and Totzke^36^ found that under autonomous driving conditions, drivers with a lower mental workload participated more frequently in non-driving-related tasks. This seems to be a behaviour characteristic of the driver’s regulation of their mental workload; however, research on the self-regulation of drivers is still rare. Future research can further explore the self-regulation of drivers’ passive fatigue, such as the timing of the regulation, the fluctuation law of the regulation, and whether regulation is a conscious strategy or an unconscious automatic adaptation process.

## Limitations

First, this study did not find a quantitative indicator of mental effort for which to prove the existence of driver self-regulation. We believe that mental effort refers to the extra effort required to maintain stable performance when the driver experiences passive fatigue. De Waard^37^ called this state-related effort. In addition to developing a wealth of external control tasks, future research should focus on drivers’ self-regulation. Second, the length of the experiment designed in this study was too short, demonstrating that the passive fatigue group drivers were still different from other groups in the sixth stage after regulation occurs. It is impossible to explore whether the driver’s self-regulation has elastic fluctuations. Future research should extend the duration of the experiment to explore whether there is a limit to the passive fatigue control scope. Third, the complex manual driving scene did not induce the driver’s active fatigue state; therefore, Hancock and Desmond’s^6^ fatigue theory is still not comprehensive. Future studies can set up a high task demand group to explore the neural mechanism of active fatigue induced by overload.

In the future, road traffic should remain in the driver-based mode with machines as the supplementary mode. While self-driving vehicles create a relaxed driving environment for drivers, it is also effortless to keep their mental workload at a lower level. An insufficient mental workload of approximately 40 min induces a passive fatigue state in the driver, which causes performance degradation and affects traffic safety. Ensuring that the driver’s workload level is stable and slow as well as avoiding passive fatigue is an intricate problem that traffic psychology must solve.

## Conclusion

Based on a comprehensive measurement of 48 drivers, we discussed the impact of driving patterns and scenarios on drivers’ mental workload. We proposed criteria for the effectiveness of passive fatigue induction and improved the driver passive fatigue theory by defining the time and critical threshold of passive fatigue. The following conclusions were drawn: (a) in automated driving under monotonic conditions, the driver’s mental workload is low; (b) when the driver is in a low-load state for approximately 40 min, passive fatigue occurs; and (c) when the driver’s EEG alpha power is above 0.000852, the driver is in a passive fatigue state.
